# Computerised prediction of healing for venous leg ulcers

**DOI:** 10.1038/s41598-022-20835-y

**Published:** 2022-10-26

**Authors:** Quoc Cuong Ngo, Rajna Ogrin, Dinesh Kant Kumar

**Affiliations:** 1 School of Engineering, STEM College, RMIT University, 124 Latrobe Street, Melbourne, VIC 3000 Australia; 2 Bolton Clarke Research Institute, Forest Hill, VIC 3131 Australia; 3 Department of Business Strategy and Innovation, Griffith University, Gold Coast, Australia

**Keywords:** Biomedical engineering, Predictive markers

## Abstract

Early prediction of delayed healing for venous leg ulcers could improve management outcomes by enabling earlier initiation of adjuvant therapies. In this paper, we propose a framework for computerised prediction of healing for venous leg ulcers assessed in home settings using thermal images of the 0 week. Wound data of 56 older participants over 12 weeks were used for the study. Thermal images of the wounds were collected in their homes and labelled as healed or unhealed at the 12th week follow up. Textural information of the thermal images at week 0 was extracted. Thermal images of unhealed wounds had a higher variation of grey tones distribution. We demonstrated that the first three principal components of the textural features from one timepoint can be used as an input to a Bayesian neural network to discriminate between healed and unhealed wounds. Using the optimal Bayesian neural network, the classification results showed 78.57% sensitivity and 60.00% specificity. This non-contact method, incorporating machine learning, can provide a computerised prediction of this delay in the first assessment (week 0) in participants’ homes compared to the current method that is able to do this in 3rd week and requires contact digital planimetry.

## Introduction

Chronic wounds are a common global health issue^1^. The most common chronic wounds are leg ulcers, where the most common underlying cause of leg ulcers is venous insufficiency^2^. Unfortunately, the prevalence of venous leg ulceration in the general population is not accurately known, but estimated at approximately 1%^2^, with prevalence increasing with increasing age^3^. Venous leg ulcers (VLU) cause a significant negative impact on the quality of life of individuals^4^ and the economic burden on individuals and the health system required to assess and manage the issue is also considerable^5^.

It is important to assess and treat chronic wounds early to ensure these are treated appropriately. Normal healing constitutes a reduction in wound area of 50% within 4 weeks^6^. Despite best practice management, over 20% of ulcers do not heal in the expected trajectory and may require additional interventions to improve outcomes^7,8^. Currently, the most readily available methods require manually monitoring of the wound area over several weeks (typically four weeks) using wound tracings, along with subjective wound characteristics. This delays the identification of wounds with abnormally delayed healing trajectory^9^ and can lead to long-term problems including amputation.

There is a need for a quick, objective, non-invasive way to ascertain the wound healing potential of chronic wounds^10^. In addition, the method must also be accurate when used by many different healthcare providers and in varied environments, as clinical care is provided in many different locations, including specialist clinics, general practice and in individuals’ homes^11–13^.

Research has established that area of the thermal image could identify the development and progression of diabetes-related foot ulcers^14^. Unfortunately, this method was unsuitable to predict the healing of VLU when used in the home environment, likely due to the ambient and individual factors which cannot be controlled^15^. An alternative is the textural analysis of thermal images, which provides information on spatial heat distribution when applied on thermal images. Given the significant change in the texture of the wounds over the healing trajectory, this method was hypothesised to identify wound healability^16^. Our previous work showed that this analysis type overcomes the ambient and other uncontrolled factors, and can predict the healing of VLU^17^.

The previous work investigated the ratio of textural features of thermal images and found that there was a significant difference between the first component of principal component analysis (PCA) of unhealed and healed ulcers over the first three weeks of presenting the ulcers^17^. While this is very useful, however, suffers because it requires revisits by the patient in week 3, and also because the wound management is delayed by 3 weeks from the first presentation. The current study builds on this early work, to ascertain whether the healing of VLU can be predicted from the first wound assessment point (i.e. week 0) alone, as opposed to using the difference between weeks 2 and 0.

The aim of this study was to overcome the previous limitations and develop a computerised method for predicting the healing of VLU from the first wound checkup, without requiring controlled lighting and ambient temperature conditions. Texture analysis was performed on the thermal images of VLU before washing and dressing. The first step was pre-processing where the wound beds were isolated from the background. Textural features were obtained and then represented by the principal components. These were classified using Bayesian neural networks into unhealed and healed wounds.

## Results

### Assessment of textural features

In this work, we analysed 64 thermal images of VLU, in which 17 wounds healed by week 12, and 47 wounds remained unhealed. These images were collected from 56 older participants. The demographic and clinical information of participants is shown in Table 1.

**Table 1:** Demographic and clinical information of participants.

	Unhealed	Healed
Number of participants	40	16
Number of VLUs	47	17
Age (mean years ± SD)	79.40 (± 13.18)	77.88 (± 9.25)
Gender (male/female)	16/24	7/9
Area of ulcers at week 0 (mean cm^2^ ± SD)	26.26 (± 49.15)	15.40 (± 54.80)

*SD* standard deviation.

**Figure 1:**
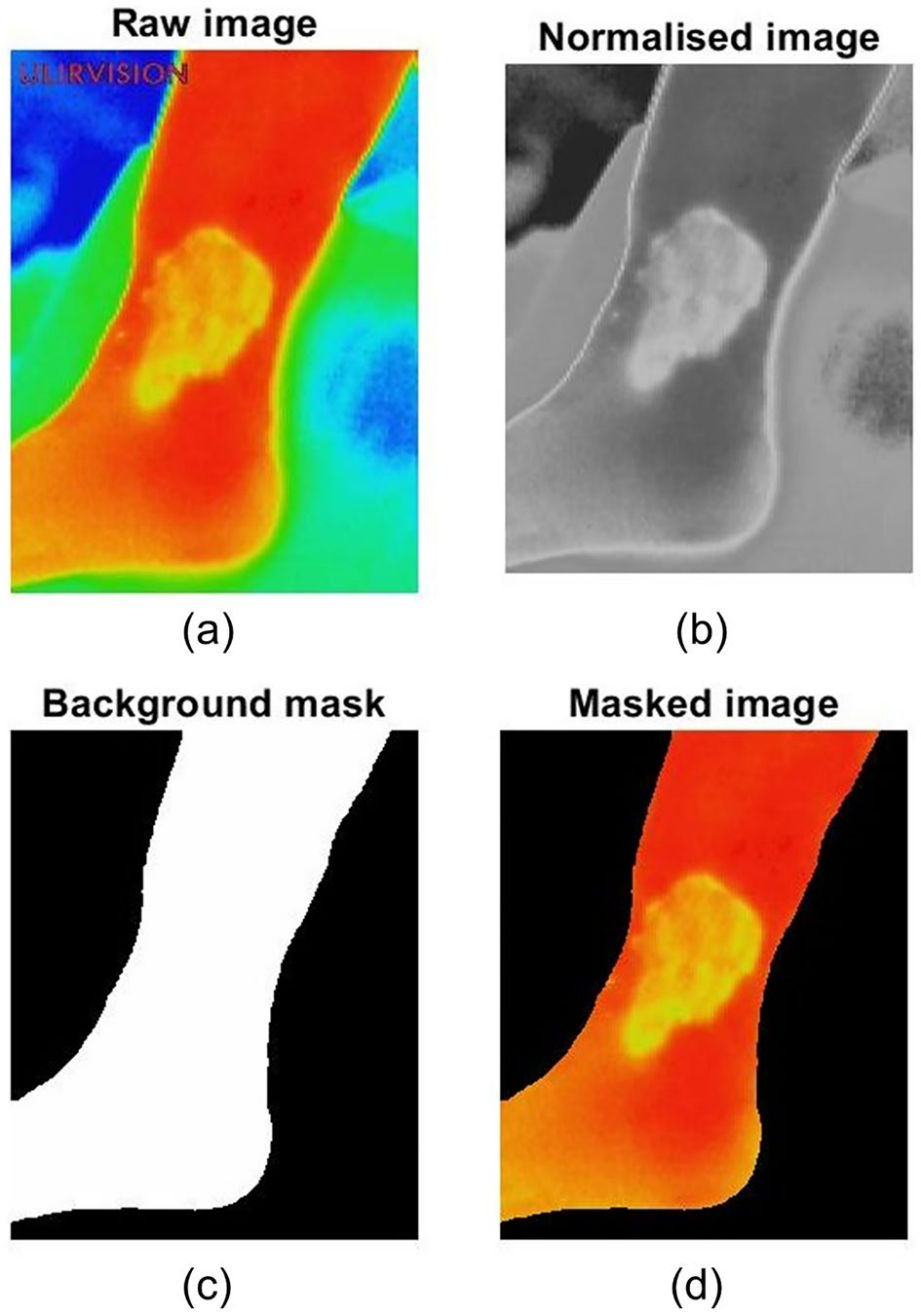
Pre-processing of a thermal image collected from a participant: (**a**) raw image; (**b**) normalised image; (**c**) background mask; and (**d**) masked image or extracted wound bed.

The thermal images of the wounds were taken in participants’ homes and there was a wide variation of surroundings and ambient conditions between the recordings. To overcome these differences, the raw image was normalised and passed through a mask. The noisy background was then removed from the raw image and the wound bed was isolated. Figure 1 shows the results of pre-processing of a representative thermal image.

**Figure 2:**
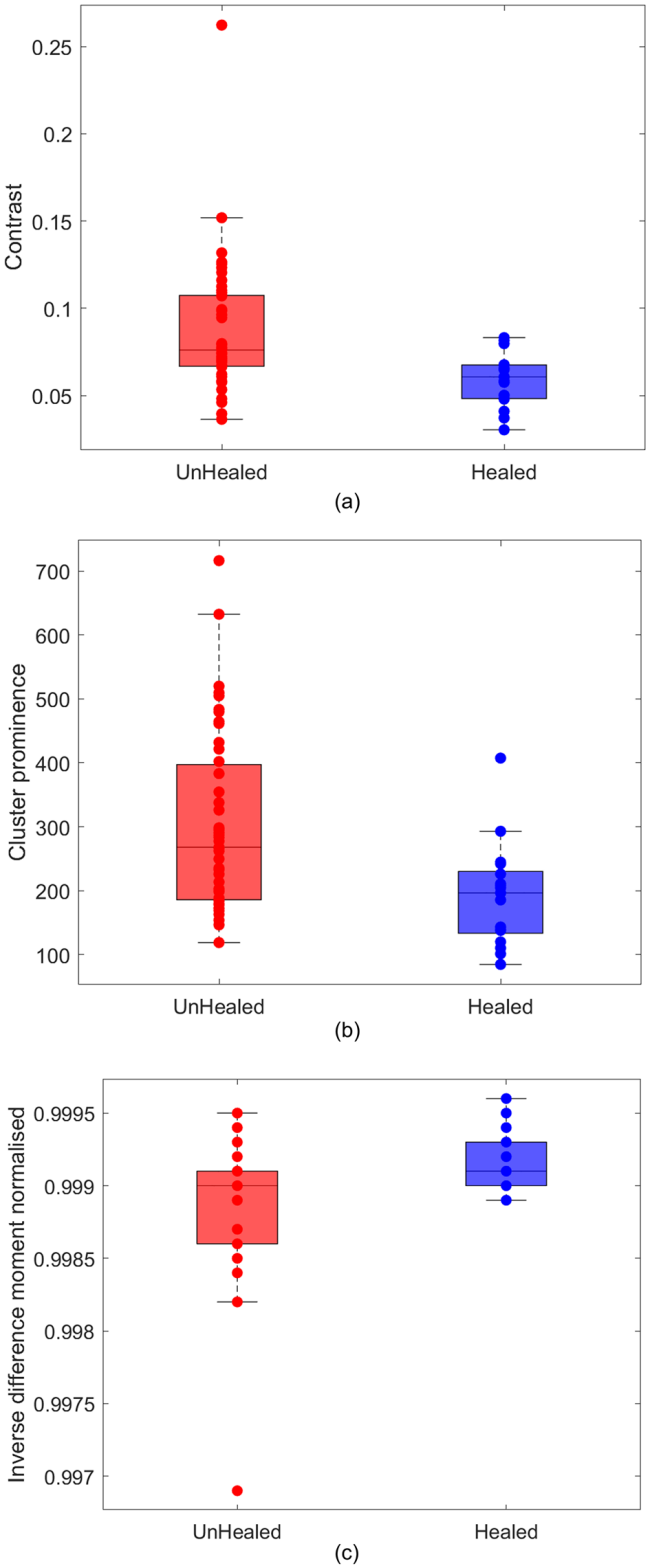
The contrast (**a**), cluster prominence (**b**), and inverse difference moment normalised (**c**) of thermal images for unhealed and healed wounds presented as boxplots and single values.

From the pre-processed images, 19 textural features were automatically extracted using proprietary software. These were grouped based on the 12-week post-presentation healing label: healed and unhealed. The median values of the features are presented in Table 2. The features were analysed and ranked based on the most significant difference between the healed and unhealed groups. The top three textural features that provided the most significant differences were contrast, cluster prominence, and inverse different moment normalised, shown as box plots in Fig. 2.

**Table 2:** Median values of textural features of unhealed and healed wounds.

Feature	Description	Unhealed	Healed
*Eng*	Energy	0.2989	0.3326
*Cont*	Contrast	0.0761	0.0607
*Corr*	Correlation	0.9863	0.9870
*sumSq*	Sum of squares	14.5656	13.3819
*sumAv*	Sum average	6.8435	6.7989
*sumVar*	Sum variance	40.2353	38.6674
*Entr*	Entropy	1.4860	1.3609
*sumEnt*	Sum entropy	1.4496	1.3360
*diffEnt*	Difference entropy	0.1714	0.1457
*imCorr*	Information measures of correlation	− 0.8772	− 0.8884
*Hom*	Homogeneity	0.9803	0.9840
*aCorr*	Autocorrelation	14.633	13.4534
*dSim*	Dissimilarity	0.0449	0.0370
*clSha*	Cluster shade	− 10.8406	− 17.6497
*clPro*	Cluster prominence	267.8896	196.5504
*maxProb*	Maximum probability	0.4221	0.4807
*idHom*	Inverse difference is homomorphic	0.9472	0.9402
*idNorm*	Inverse difference normalised	0.9953	0.9962
*idmNorm*	Inverse difference moment normalised	0.9990	0.9991

Figure 2a shows that the contrast in thermal images of the unhealed wounds was significantly higher than those of healed wounds (P = 0.001). Figure 2b shows significantly higher cluster prominence calculated from unhealed ulcers compared to healed cases (P = 0.002). Conversely, Fig. 2c shows that the inverse difference moment normalised indices of unhealed wounds were significantly lower than healed wounds (P = 0.001).

**Figure 3:**
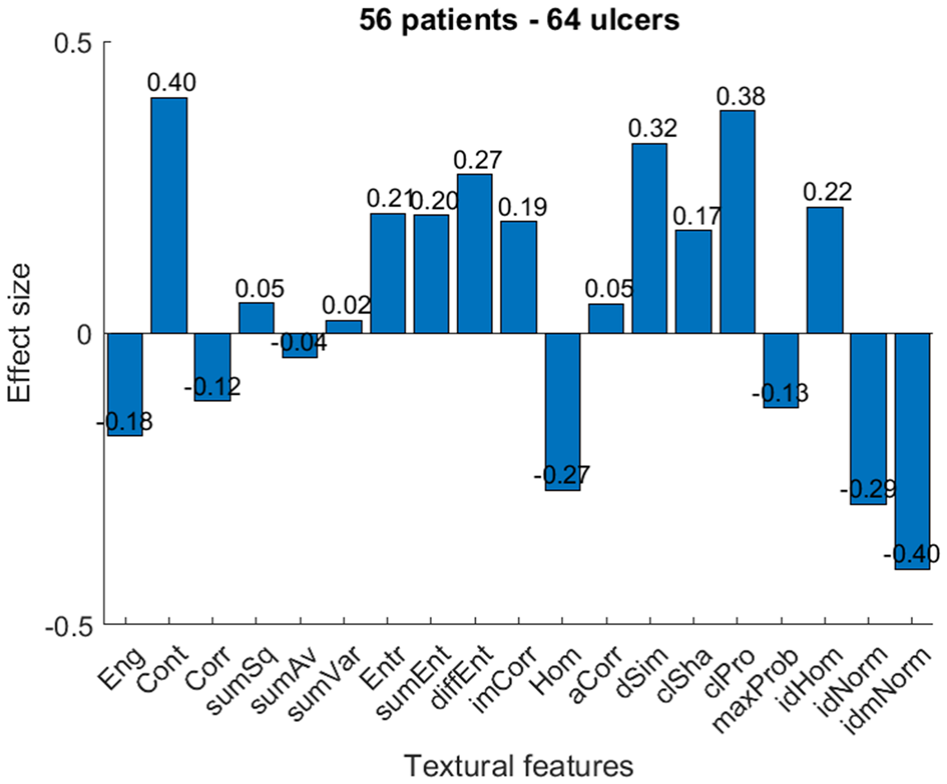
Effect size of 19 textural features obtained from 64 thermal images of 56 patients.

**Figure 4:**
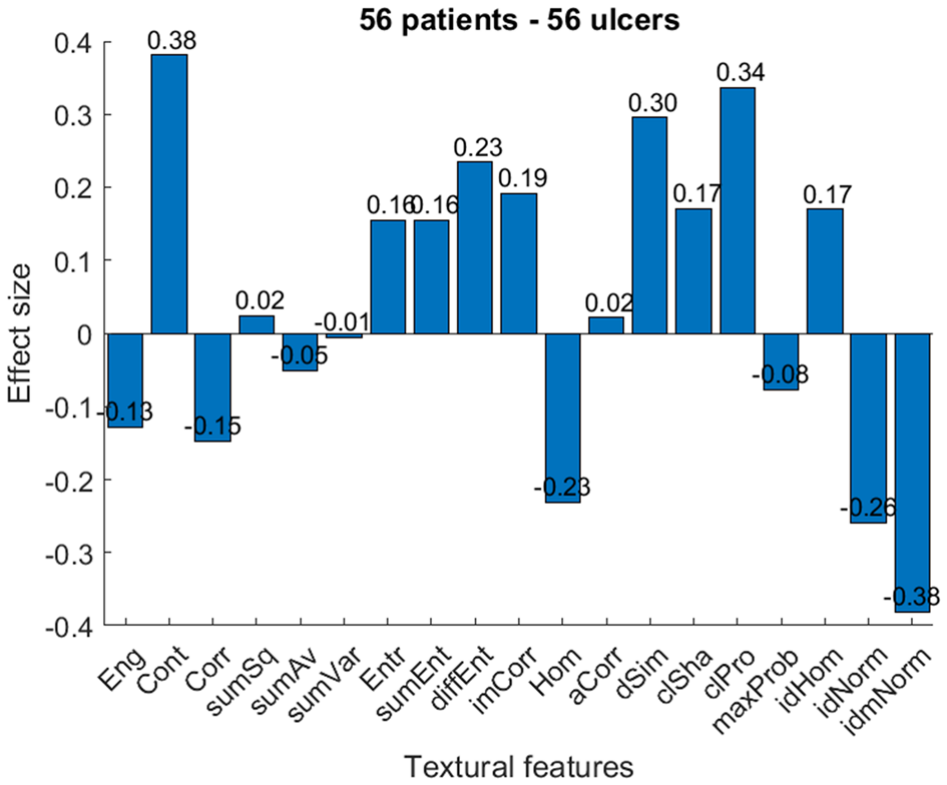
Effect size of 19 textural features of 56 images of 56 patients-when only one ulcer from each patient is considered.

The effect sizes of different textural features of 64 ulcers are shown in Fig. 3. Eight out of nineteen textural features had an effect size greater than 0.2. Among these, the feature of contrast provided the largest effect size when comparing unhealed to healed wounds. Figure 4 presents the effect size when only one ulcer from each patient is considered (i.e. 56 ulcers). It is worth noticing that there were no significant differences.

### Classification of wounds

The dataset of this study consists of vectors of 19 texture features. The dimension of the data was reduced using PCA. For classification purposes, 70% of the data were used for training and the balance for testing. The division of data was conducted randomly and with 20 repetitions.

**Figure 5:**
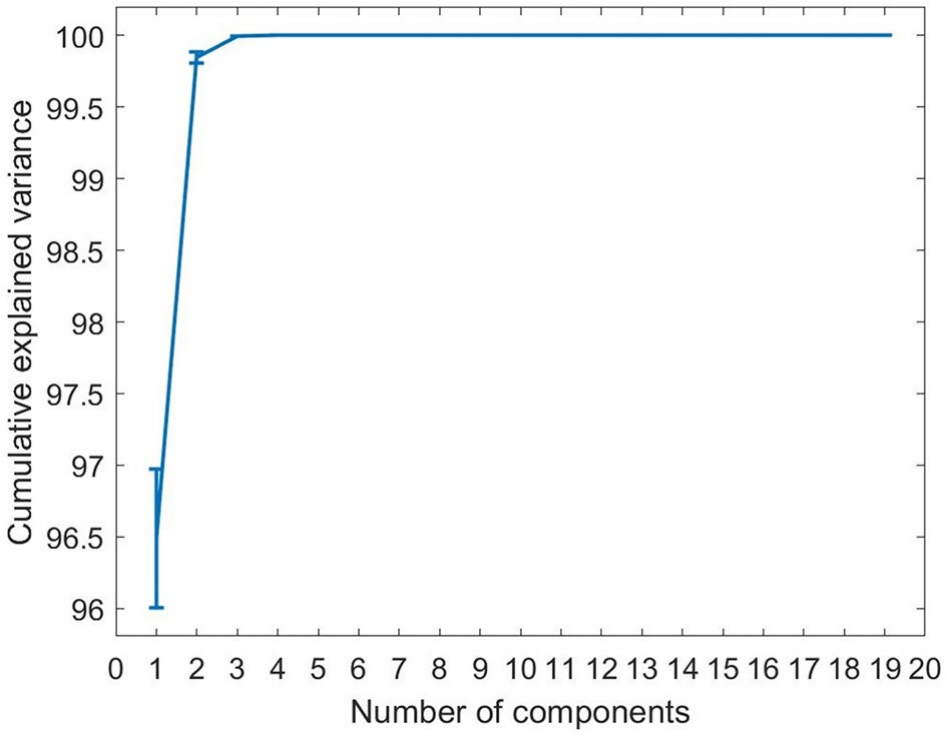
The cumulative explained variance plot of principal component analysis from 19 components.

The cumulative explained variance obtained using the training set is shown in Fig. 5. This figure shows that the 19 textural features can be represented by three PCA components (99.99%), where the first component contributed to 96.44% of the variance. The pattern of three PCA elements for each image was the input to the Bayesian network classifier. Tangent sigmoid functions were used for the hidden layer transmission. There was one output node that was activated by a sigmoid function.

**Figure 6:**
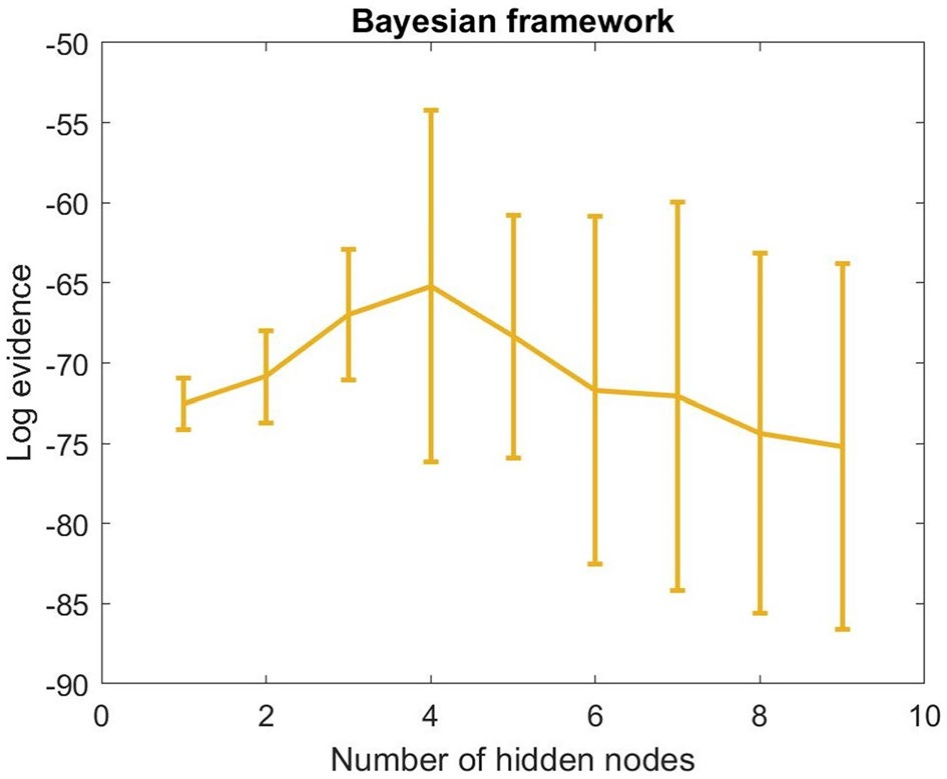
Log evidence was obtained from the neural networks with a different number of hidden nodes using a Bayesian framework.

Figure 6 presents the log evidence computed from the Bayesian framework using the training data. The number of hidden nodes was varied from 1 to 9. The figure shows that the network with 4 hidden nodes provided the highest log evidence. Thus, this network was selected for labelling the ulcers as healing ulcers and unhealing ulcers.

In the testing phase, feature vectors of a test set were projected to the PCA matrix of the training set to produce the PCA pattern vectors for the test data. The average classification accuracy for the healing labels obtained over 20 repetitions is presented in Table 3. The average correct detection rate of unhealed wounds was set at 75.10% in the training data, and 71.43% for the test data set. The best test classification results achieved were 78.57% sensitivity and 60.00% specificity.

**Table 3:** Classification results of healing status at week 12 using week 0 information (%).

	Training		Testing	
	Sensitivity	Specificity	Sensitivity	Specificity
Mean (SD)	75.10 (7.61)	64.75 (4.00)	71.43 (11.35)	60.00 (17.17)
Best	79.00	67.00	78.57	60.00

*SD* standard deviation.

**Figure 7:**
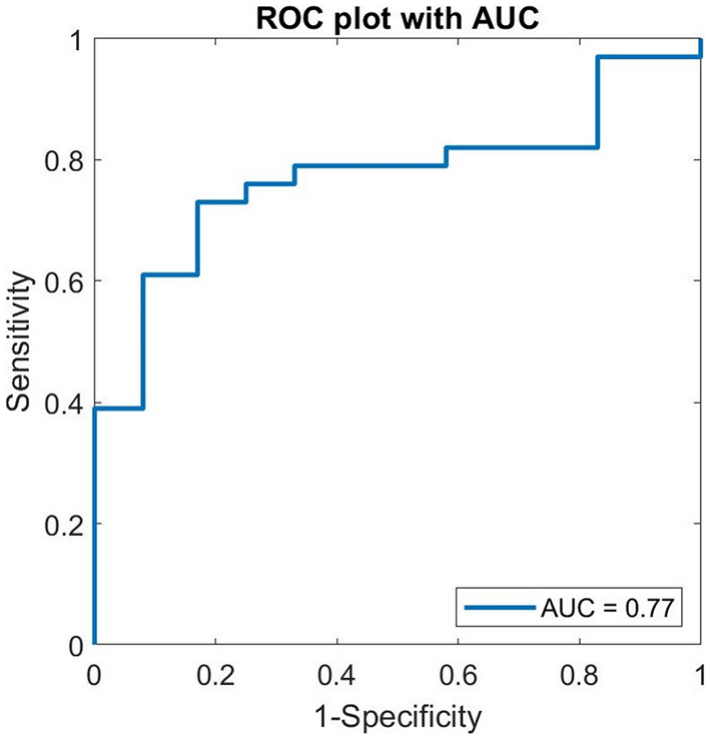
ROC curve and AUC were obtained from the optimal model for differentiating between unhealed and healed wounds.

The receiver operating characteristic (ROC) curve of the training set corresponding to the best classification result is shown in Fig. 7. The area under the curve (AUC) is 0.77, which is considered a good value for this application^18^. From the curve, we can choose a threshold to maximise the correct detection rate of unhealed wounds (sensitivity) or of healed wounds (specificity).

The proposed framework was written in MATLAB language (MATLAB 2019b, The MathWorks, Natick, MA, USA). We used MATLAB's built-in time functions on the hardware with Intel Core i5-1035G7 CPU 1.20 GHz processor and 8 GB RAM for the estimation of time consumption. The average of the total computation time, including for reading, processing, and classification of an image was 0.39 ± 0.02 s.

## Discussion

This study identified that using textural features of thermal images taken at the first assessment (week 0) of the VLU can predict whether the wound would heal by week 12. The analysis was undertaken using proprietary software that performed automatic pre-processing of the images followed by feature extraction, dimensionality reduction, and classification. The classification performance was obtained by dividing the dataset into 70% training and 30% test data, with 20 repetitions. The results show that the software was able to predict the healing label in week 0 with an average of 71.43% sensitivity and 60% specificity.

Currently, the prediction of healing for chronic VLU (and other chronic wounds) is undertaken by measuring the wound area using digital planimetry and reviewing the area after at least 2 and often up to 4 weeks^19^. This method delays the initiation of adjuvant therapies for unhealing wounds by 4 weeks. Further, it also requires physical contact with the wound, potentially increasing the risk of infection. A method that is non-contact, and which can provide this information at the first appointment can support the early implementation of appropriate management to enable improved healing trajectories^20^.

Thermal image-based wound assessment to predict healing has been reported previously, with limited success. Using thermal imaging for measuring wounds was found to require controlled lighting and ambient temperature, and the results were dependent on the colour and texture of the individuals' skin^15,21^. This limitation was overcome by measuring the change in the thermal images over 2 consecutive weeks^17^. While an improvement from the typical 4 weeks, nevertheless, this still requires a delay of 2 weeks.

Unlike earlier attempts, we investigated the texture of the image of week 0 and did not consider the change of the images over multiple weeks. The results show that the textural analysis of the first presentation itself has sufficient information to predict the week 12 wound healing status. This is a significant improvement because it can identify those patients requiring additional treatments at the first visit. Further, the data used was from images that were taken during home visits, without controlled lighting or temperature conditions, showing that these images do not require to be captured in controlled conditions. This is important because people with VLU receive care in a wide range of places such as general practice, other clinical settings and in the homes of people receiving home nursing^11^.

The contrast feature of the thermal images of unhealed wounds had higher local variations (0.0761 vs. 0.0607) whereas the cluster prominence and cluster shade features showed that the thermal images of unhealed wounds were more asymmetric than healed wounds (267.8896 vs. 196.5504). The *idmNorm* feature is a measure of homogeneity. This study found that the thermal images of healed wounds had smaller grey tone differences (i.e. this feature of healed wounds was greater than unhealed wounds, 0.9991 vs. 0.9990).

It can be seen from Fig. 2 that there were outliers in the extracted features. Although PCA is sensitive to outliers, outliers were not removed before PCA so that it reflects real-world scenario. The intention of the proposed algorithm is for machine based analysis and to be used in a wide range of settings where the existence of outliers may be unavoidable.

There are two major limitations in the current study. Firstly, the number of healed wounds was relatively small compared to unhealed wounds resulting in an unbalanced dataset. However, the potential usefulness of the proposed algorithm for the prediction of healing status is confirmed with evidence from the classification results averaged over 20 repetitions. In addition, the performance of the method used in the current study is significantly higher than methods extracting wound areas from thermal images^15^. The other limitation is that it only investigated older people.

Future research should focus on improving the predictive accuracy and customising this method to incorporate this assessment into clinical practice on a wider pool of participants and in a variety of settings. This will require an algorithm embedded within an app that will automatically generate the wound healing prediction after taking a digital image. This could be done by transferring the framework used in this study to a mobile phone app. To support feasibility in clinical settings the collection of thermal images will be enabled using commercial thermal imaging cameras, which can be attached directly to a mobile phone.

## Methods

### Study protocol

This study was conducted in accordance with the ethics approval from the Human Research and Ethics Committee of Bolton Clarke (Project number: 194) and RMIT University (BSEHAPP 21-15). The protocol and data of the study have been reported earlier^17^. Participants were recruited from the northern region of Melbourne’s metropolitan area. The criteria for participant recruitment are presented in Table 4. All participants provided written informed consent to take part in the study.

**Table 4:** Participant recruitment criteria of the study of venous leg ulcers (VLU).

Inclusion criteria	Exclusion criteria
Adults Lived in the catchment area of the nursing service Had a VLU diagnosed from clinical indications Either an Ankle Brachial index between 0.8 and 1.2, or a duplex scan indicating no arterial involvement Available for consecutive weekly visits over the study period	Non-venous wound primary diagnosis And/or if the individual would heal within the study period, based on wound either almost or fully epithelialized in less than two weeks

Thermal images of VLU were collected weekly in the first three weeks by a trained research nurse at participants’ homes using the handheld ULRIvision TI160 (Zhejiang Ulirvision Technology Co., Ltd). This device produces images with a resolution of 160 × 120. We also collected digital planimetry data using VISITRAK (Smith & Nephew Healthcare Ltd.). The emissivity of the device was set equal to that of clean human skin (i.e. 0.98). The two-dimensional surface area data of ulcers obtained from the device were used as a reference in this study.

The healing status of wounds was monitored until week twelve. Wounds that healed on or before week 12 were labelled as “healed”, and those that did not heal were labelled as “unhealed”.

### Pre-processing

Thermal images were pre-processed automatically in three steps: image normalisation, background mask creating, and wound bed extracting. Firstly, the company logo of the thermal imaging device was removed from the images. We then converted the colour map from the RGB (red, green, and blue) scale to the greyscale. Secondly, a threshold was applied to discriminate the wound bed from the image background. Consequently, we created a background mask. The mask was enhanced using morphological operations, including hole filling and image eroding^22^. Finally, the wound bed was extracted using the enhanced mask.

### Textural analysis

Textural features describe the spatial distribution of grey tones in an image. This method has been used extensively in a variety of biomedical applications such as the detection of skin tumours^23^, classifying breast ultrasound^24^. In this study, texture information of the thermal images was extracted using a grey level co-occurrence matrix (GLCM)^25^. The texture information is specified by a matrix of angular relationship (*α* = 0°, 45°, 90°, 135°) and distance (*d* = 1, 2, 3, …) between two neighbouring pixels. The names of textural features used in this work are presented in Table 2. The detail of these features can be found at^25–27^.

A range of values of the angle and distance was tried, and the ones that provided the best results were selected. In this study, we used the angle of 90° and the distance of 1 to calculate the textural features of thermal images at week 0.

### Statistical analysis

In this study, we performed Mann–Whitney U tests to identify the level of significance of the difference in textural features between unhealed and healed ulcers in week 0 because the data distribution was not normal. Features having P-values less than 0.05 were considered to be statistically significant.

### Principal component analysis

Principal component analysis can help reduce the dimension of a dataset while preserving as much original information as possible^28^. Using PCA, we transformed the textural features into principal components. Each principal component contributes a proportion of the total variation in data. The first principal component accounts for the largest variance in the data. To calculate how many components are required to represent the data, we looked at the cumulative explained variance. In this study, we found that the first three components were sufficient to describe the thermal image dataset.

70% of the data were used as a training set to extract principal components and the balance 30% was used for testing. The division of data was conducted randomly and repeated over 20 times. The PCA scores of the test set were computed by projecting the test data set on the principal components obtained from the training set. The first three principal component scores of the train and test sets were then exploited as input for a Bayesian neural network in the training and testing phases.

### Bayesian neural network

To distinguish between healed and unhealed wounds, we trained and tested a Bayesian neural network (BNN) using the extracted principal components. The Bayesian framework can improve the generalisation property for normal neural networks, in which the input is a limited or noisy dataset.

A typical Bayesian neural network has a feedforward structure: one input layer, one hidden layer, and one output layer. The network in this study had a 3-node input, which is the first three principal components. The optimal number of hidden nodes was estimated using the Bayesian evidence framework^29–32^. In the framework, values of log evidence were calculated for a different number of hidden nodes using the training data. The network weights and biases were assumed to have a Gaussian probability distribution and were initialised randomly.

The network that provided the highest log evidence was selected for the study. The output node provided information about the healing status (unhealed vs. healed). On minimising the cost function of network training, the weight decay algorithm^33,34^ was used in this study. The performance of the classifier was presented through a pair of sensitivity and specificity, and a receiver operating characteristic curve.

## Data Availability

The de-identified data is available upon request to the corresponding author, dinesh.kumar@rmit.edu.au.
